# Delivery of Rice Gall Dwarf Virus Into Plant Phloem by Its Leafhopper Vectors Activates Callose Deposition to Enhance Viral Transmission

**DOI:** 10.3389/fmicb.2021.662577

**Published:** 2021-05-05

**Authors:** Ge Yi, Wei Wu, Taiyun Wei

**Affiliations:** State Key Laboratory of Ecological Pest Control for Fujian and Taiwan Crops, Fujian Agriculture and Forestry University, Fuzhou, China

**Keywords:** phloem-limited virus, rice leafhopper, insect feeding behavior, callose deposition, callose synthase genes

## Abstract

Rice gall dwarf virus (RGDV) and its leafhopper vector *Recilia dorsalis* are plant phloem-inhabiting pests. Currently, how the delivery of plant viruses into plant phloem *via* piercing-sucking insects modulates callose deposition to promote viral transmission remains poorly understood. Here, we initially demonstrated that nonviruliferous *R. dorsalis* preferred feeding on RGDV-infected rice plants than viruliferous counterpart. Electrical penetration graph assay showed that viruliferous *R. dorsalis* encountered stronger physical barriers than nonviruliferous insects during feeding, finally prolonging salivary secretion and ingestion probing. Viruliferous *R. dorsalis* feeding induced more defense-associated callose deposition on sieve plates of rice phloem. Furthermore, RGDV infection significantly increased the cytosolic Ca^2+^ level in rice plants, triggering substantial callose deposition. Such a virus-mediated insect feeding behavior change potentially impedes insects from continuously ingesting phloem sap and promotes the secretion of more infectious virions from the salivary glands into rice phloem. This is the first study demonstrating that the delivery of a phloem-limited virus by piercing-sucking insects into the plant phloem activates the defense-associated callose deposition to enhance viral transmission.

## Introduction

Numerous devastating insects feeding on the phloem and viral pathogens living in the phloem cause significant economic losses in major crops. Piercing-sucking insects, including leafhoppers, planthoppers, aphids, and whiteflies, horizontally transmit viral pathogens *via* saliva to plant phloem (Jia et al., 2018). For instance, leafhoppers, a large insect family with approximately 22,000 described species, transmit numerous phloem-limited plant viruses (Wang et al., 2017). Insect vector behavior has profound ecological and evolutionary implications on transmitted viral pathogens; thus, viral infection would alter the vector feeding behavior to facilitate its own transmission (Hurd, 2003; Angelella et al., 2018). Several animal-infecting viruses change the ingestion behavior of their insect vectors by increasing their biting rates. For instance, there are numerous reports on increased biting rates in mosquitoes infected with La Crosse virus, Semiliki forest virus, Rift Valley fever virus, and Dengue virus (Hurd, 2003). Similarly, plant viruses also directly influence vector feeding behavior to facilitate their transmission (Mauck et al., 2018). Several reports show that infection of insect vectors with tomato spotted wilt virus (TSWV), tomato yellow leaf curl virus, rice dwarf virus, and southern rice black-streaked dwarf virus (SRBSDV) is associated with increased insect feeding frequency and prolongs salivation period (Maris et al., 2004; Xu et al., 2014; Lei et al., 2016; Tan et al., 2017; Wang et al., 2018; Lu et al., 2019). Moreover, vector feeding behavior is correlated with sex and vector biotypes. For instance, male thrips are more likely to acquire and spread TSWV than female thrips (Stafford et al., 2011). However, the mechanisms underlying the complex association between phloem-limited plant viruses with insect vectors remain elusive.

Phloem is a long-distance transport system with highly evolved vascular tissue comprising sieve elements, companion cells, and parenchyma cells (Heo et al., 2014). Phloem is also a habitat for piercing-sucking insects (Heo et al., 2014). The stylet of piercing-sucking insects often takes a long route in the intercellular spaces (apoplast) to reach the phloem; and thus, the insects secrete watery saliva proteins into phloem cells to interfere with defense-associated callose deposition at sieve plates (Jiang et al., 2019). Insect vectors deliver viral pathogens into the sieve cells *via* saliva (Wei and Li, 2016). Callose-associated sieve plate occlusion represents a potentially unique phloem defense strategy against viral pathogens and insects (Hao et al., 2008). Sieve plate occlusion by callose sealing is likely depend on Ca^2+^ accumulation in sieve cells (Hao et al., 2008). However, piercing-sucking insects secrete Ca^2+^-binding protein (CBP) in watery saliva to prevent sieve cell occlusion in plant phloem (Will et al., 2007; Atamian et al., 2013). Although the insect-mediated delivery of viruses into the sieve cells potentially modulates the defense-associated callose deposition of plant phloem, the underlying mechanism remains unknown.

In Asian rice-growing countries, rice gall dwarf virus (RGDV), a plant reovirus, causes epidemic disease and significant rice yield losses (Mao et al., 2019). RGDV is mainly transmitted by green rice leafhopper *Recilia dorsalis* (Hemiptera: Cicadellidae) in a persistent-propagative manner (Liao et al., 2017; Mao et al., 2019). During feeding, *R. dorsalis* ingests the phloem sap of RGDV-infected rice plants *via* the stylet and transmits the virions into healthy plants through saliva secretion (Mao et al., 2017). Previous studies found that *R. dorsalis* infection with RGDV prolongs the nymph development, shortens adult longevity, and reduces the survival rates of nymphs and female fecundity (Chen et al., 2016). Such findings imply that RGDV infection reduces the fitness of *R. dorsalis* and enhances viral transmission (Chen et al., 2016). However, the mechanism by which RGDV impacts the feeding behavior of *R. dorsalis* to enhance viral transmission is unclear. In this article, we explore how RGDV infection modifies leafhopper feeding behaviors to facilitate viral transmission. We demonstrate that viruliferous leafhopper feeding induces the deposition of more defense-associated calloses on sieve plates of rice phloem than nonviruliferous control. Viruliferous *R. dorsalis* feeding significantly elevates cytosolic Ca^2+^ level in rice phloem cells, causing substantial callose deposition. Thus, the delivery of RGDV by *R. dorsalis* into rice phloem potentially activates callose deposition to enhance viral transmission.

## Materials and Methods

### Virus, Insect Vector, and Antibody

The RGDV isolate was collected from infected rice fields in Xingning, Guangdong, China. The nonviruliferous *R. dorsalis* population was collected from rice fields in Xingning, Guangdong Province, China. To acquire viruliferous *R. dorsalis*, nonviruliferous nymphs were fed on RGDV-infected TN1 rice seedlings for 2 days and then transferred to rice seedlings, as previously described (Chen et al., 2016). Rice seedlings were replaced daily for 12 days. RT-PCR assay was applied to validate RGDV infection of *R. dorsalis*. The F1 progeny of viruliferous *R. dorsalis* was reared for subsequent experiments. Rabbit polyclonal antibody against RGDV-encoded P8 protein was prepared as previously described (Mao et al., 2019). Polyclonal antibody was conjugated directly to fluorescein isothiocyanate (FITC) following the manufacturer’s instructions.

### Host Plant Selection Preference of *R. dorsalis*

Healthy and RGDV-infected rice seedlings were planted in plastic pots (*D* = 15 cm), and put in the same cage (*L* = 50 cm, *W* = 35 cm, and *H* = 40 cm) with two side-windows (*D* = 5 cm) of nylon mesh mid-cylinder. Nonviruliferous or viruliferous insects (*n* = 30) were transferred into each cage. The number of insects on RGDV-free or RGDV-infected rice plants was recorded at 1, 3, 6, 12, 24, 48, and 72 h. The experiments were conducted in three biologically independent replicates.

### Electrical Penetration Graph Recordings

A day before the electrical penetration graph (EPG) assay, rice seedlings were transplanted into turf soil-filled plastic pots. A trematode tube was used to suck the insect into a glass tube and then put on CO_2_ under anesthesia for 10 s. The dorsal thorax of the insect was connected to one end of a gold wire (*D* = 20 μm, *H* = 10–15 cm) using a water-soluble silver glue. The glue was dried for about 2–3 min to ensure a tight junction with the insect. The wired insect was connected to the EPG probe *via* a copper nail. The probe was connected to the amplifier, and then the tested insects were placed on the rice stem. A copper wire (*D* = 2 mm and *H* = 10 cm) was inserted into the potting soil vertically and connected to another amplifier. Subsequently, the electrical EPG signals were amplified and digitized using a converter. The STYLET software (Wageningen University, Wageningen, Netherlands) was employed for data acquisition. Data for each insect were continuously recorded for 5 h. Using the random design method, we recorded at least 20 biologically independent replicates for each insect.

### Plant Odor Selection Preference of *R. dorsalis*

A glass Y-tube olfactometer was applied to test plant odor selection preference of *R. dorsalis* following the method described by Signoretti et al. (2012) with minor modifications. The Y-shaped tube (4-cm inner diameter) comprised a central tube (10 cm long) and two arms (18 cm long, offset by 75°) connected to the living plant bag. The airflow was guided through an activated carbon purifier and a distillation water bottle before entering the odor source to purify the air and increase the humidity. A flow meter was used to calibrate the airflow in the olfactometer (500 ml/min) at the end of each arm. Light was introduced into the Y-tube by placing a 30-W filament lamp 30 cm above it. The semitransparent paper was used to cover the lower part of the lamp. Next, *R. dorsalis* (macropterous adults, starved for 2 h before testing) was individually placed in the central tube. Each insect was observed for 5 min. The preference of nonviruliferous or viruliferous *R. dorsalis* adults to healthy or RGDV-infected rice plants was assessed. Thirty *R. dorsalis* individuals were tested in each experimental group, and each experiment was repeated five times.

### Evaluation of the Intracellular Cytosolic Ca^2+^ Variation

To establish the intracellular cytosolic Ca^2+^ variation of rice plants, acetoxy-methyl ester of Fluo-3 (Fluo-3 AM) was applied as the Ca^2+^-sensitive fluorescent indicator (Ye et al., 2017). Briefly, Fluo-3 AM (stock solution in dimethyl sulfoxide; Molecular Probes, Eugene, OR, United States) was diluted in 50 mM MES buffer (pH 6.0) containing 0.5 mM calcium sulfate and 2.5 μM 3-(3,4-dichlorophenyl)-1,1-dimethylurea (Sigma-Aldrich, Steinheim, Germany) to a final concentration of 5 μM. Rice leaves (two-leaf stage) were confined within a self-made leaf clamping cage (diameter 3 cm and height 5 cm) with 10 viruliferous or nonviruliferous *R. dorsalis*, separately. Infected leaf parts were harvested 1, 3, and 6 h post infestation, followed immediately by a 30-min incubation in 5 μM Fluo-3 AM solution (500 μl) as described above. Subsequently, the leaves were mounted on Leica TCS SP5 inverted confocal microscope and examined at 488 nm excitation wavelength. Micrographs were taken under identical conditions. The experiment was prepared in triplicate.

### Histological Examination of Callose Deposition

Rice leaves (two-leaf stage) were inoculated with 10 insects for 6 h, and then cut into 0.3–0.5 cm pieces and placed in freezing glue. Samples were sliced into 10-μm sections using Leica CM1900 frozen ultrathin slicer with a microtome and fixed in microscope slides, followed by overnight incubation in 100% ethanol solution. Thereafter, sections were stained with 0.1% aniline blue in 0.15 M K_2_PHO_4_ for 30 min and examined under an ECLPSE Ts2R-FL fluorescence microscope (Nikon). Micrographs were taken under identical conditions for all the samples. Sieve plates with bright blue fluorescence were recorded as callosic plates. For each sample, the area of callose in sieve plates was measured for 46 cross-sections.

### Gene Expression of Callose Synthase and Hydrolase

Total RNA was extracted using TRIzol Reagent (Invitrogen) from the leaf sheaths of rice plants sampled at a specified time post-*R. dorsalis* infestation. The RNA was reverse transcribed into cDNA using Thermo Scientific Revert Aid Reverse Transcriptase (Thermo). Reference gene Actin (accession number AB047313) was used for normalization. Primers were synthesized according to Yang et al. (2017) as follows: OsGSL1-F: 5'-TGAGGACCTGCCACGATT-3'; OsGSL1-R: 5'-CACGCTGATTGCGAACAT-3'; Gns5-F: 5'-TTGCGGCCATTCCTACAGT-3'; and Gns5-R: 5'-TGGTGAGGGCGATGCTTG-3'. The reference gene was synthesized according to Du et al. (2009) as follows: Actin-F: 5'-CAGCACATTCCAGCAGAT-3'; and Actin-R: 5'-GGCTTAGCATTCTTGGGT-3'. The specificity and efficiency of each primer were verified through standard curve analysis of a 10-fold cDNA dilution series. Relative levels of gene expression were estimated *via* the 2^−△△Ct^ (cycle threshold).

### Immunofluorescence Staining of Semi-Thin Sections

Hand-cut tissues (about 30–50 mm thickness) of the leaf sheaths of rice plants were collected at a specific time post-*R. dorsalis* infestation. They were processed into 10–20-μm-thick sections using a cryostat (Leica CM1900). Tissue sections were fixed with 4% paraformaldehyde at room temperature for at least 12 h and permeabilized in 4% Triton X-100 for 6 h. Thereafter, tissues were immunolabeled with RGDV P8 antibody conjugated to FITC (Sigma). The fluorescent signals were visualized using a Leica TCS SP5II confocal microscope.

### Electron Microscopy

Virus-infected rice plants were sampled and fixed with 2.5% glutaraldehyde for 12 h at 4°C. The tissues were then fixed with 1% OsO4, dehydrated in a gradient series of ethanol from 50 to 100%, and embedded in Spurr resin (SPI Ltd.). Ultrathin sections were examined under a transmission electron microscope (HITACHI, H-7650).

### Statistical Analysis

All data were analyzed in SPSS (version 17.0; SPSS). Percentage data were arcsine square-root transformed before the analysis. LSD multiple comparison method was applied to compare the viruliferous and nonviruliferous groups. Multiple *t*-test was used for host plant selection preference analysis. Two-way ANOVA was applied for the analysis of nonviruliferous and viruliferous *R. dorsalis* odor selection preference on RGDV-free and RGDV-infected rice plants. One-way ANOVA was used to evaluate nonviruliferous and viruliferous *R. dorsalis* feeding behavior. The significant differences in callose area, gene expression levels, and the number of feeding sites were examined using the student’s *t*-test. After analysis, original data were presented in texts, figures, and tables.

## Results

### Viral Infection Affects the Host Preference of *R. dorsalis*

**Figure 1:**
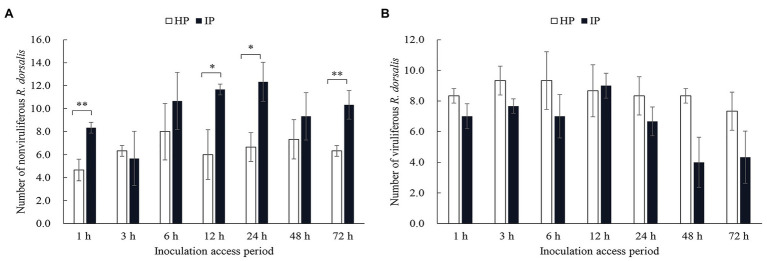
Nonviruliferous *Recilia dorsalis* preferred to feed on infected rice plants. Selective preference of nonviruliferous **(A)** or viruliferous **(B)** *R. dorsalis* adults to rice gall dwarf virus (RGDV) infected or healthy rice plants. The histogram bars show the number of insects feeding on healthy or RGDV-infected rice plants. HP, healthy plant; IP, infected plant. Significant differences in gene expression are denoted as ^*^(*p*, 0.05) or ^**^(*p*, 0.01); multiple *t*-tests.

Selective preference of viruliferous or nonviruliferous *R. dorsalis* adults to RGDV infected or healthy rice plants was recorded for 3 days after insect release. We observed that nonviruliferous *R. dorsalis* preferred to feed on RGDV-infected plants over RGDV-free rice plants from 12 h (Figure 1A). However, the preference of viruliferous *R. dorsalis* to infected and healthy rice plants was not significantly different (Figure 1B).

**Figure 2:**
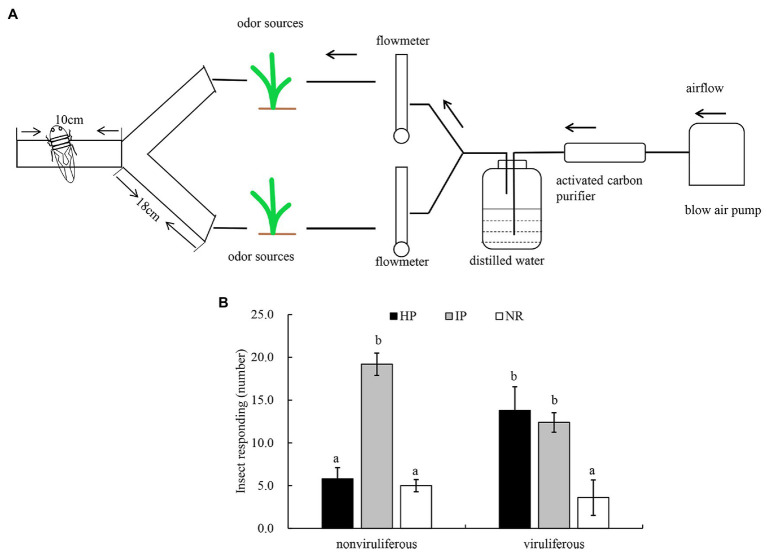
The effect of odors emitted by virus-infected rice plants on the feeding tendency of *R. dorsalis*. **(A)** Y-tube olfactometer diagram. **(B)** Selective odor preference of viruliferous or nonviruliferous *R. dorsalis* adults to RGDV infected or healthy rice plants. Each experimental group comprises 30 adults and is repeated five times in total. Viruliferous, viruliferous *R. dorsalis*; nonviruliferous, nonviruliferous *R. dorsalis*; HP, healthy plant; IP, infected plant; NR, no responding. Bars represent means ± SE.

We then employed the Y-tube olfactometer to test the effect of odors emitted by healthy or virus-infected rice plants on the feeding tendency of *R. dorsalis* (Figure 2A). We observed that nonviruliferous insects (approximately 64.0%) were largely attracted to RGDV-infected rice plants over healthy rice plants (*F* = 18.374, *df* = 4, *p* = 0.0017; Figure 2B). No significant difference was found between males and females (*χ*^2^ = 0.0189, *df* = 1, *p* = 0.21; Table 1). The odor selection preference of viruliferous individuals to either infected or healthy plants revealed that about 46.0% were attracted to healthy plants, whereas 42.0% were attracted to the infected plants. No significant difference was found between infected and healthy rice plants (*F* = 0.318, *df* = 4, *p* = 0.67; Figure 2B) or males and females (*χ*^2^ = 0.0234, *df* = 1, *p* = 0.32; Table 2) for viruliferous insects odor preference test. Thus, the above results suggest that odor emitted by infected rice plants could attract nonviruliferous *R. dorsalis* and promotes virus acquisition by the insects.

**Table 1:** Selective responses of viruliferous and nonviruliferous *R. dorsalis* adults to volatiles from RGDV-infected and uninfected rice plants.

Replicate	Nonviruliferous			Viruliferous		
	HP (♀/♂)	IP (♀/♂)	NR (♀/♂)	HP (♀/♂)	IP (♀/♂)	NR (♀/♂)
1	6/1	7/11	2/3	7/5	6/8	2/2
2	3/2	9/11	3/2	9/8	4/7	2/0
3	4/2	7/11	4/2	9/6	5/7	1/2
4	1/3	12/9	2/3	6/4	6/7	3/4
5	2/5	9/10	4/0	6/9	9/4	0/2
Total	16/13	44/52	15/10	37/32	30/33	8/10
Average (♀+♂)	5.80 ± 0.58^a^	19 ± 1.30^b^	5.0 ± 0.71	13.80 ± 1.24^b^	12.60 ± 0.51^b^	3.60 ± 0.93^a^

HP: healthy plant; IP: infected plant; NR: not responding; mean ± SE is the mean of five replicates and SE. Values in the same row at the same status (nonviruliferous or viruliferous) that are followed by different letters show significant differences at *p* < 0.05 level (LSD test).

**Table 2:** Statistical analysis of selective responses of viruliferous and nonviruliferous *R. dorsalis* to volatiles from virus-infected and uninfected rice plants.

*p*^a^		
*R. dorsalis*	Rice	R. dorsalis*Rice
0.387	<0.001	<0.001

a Parameter codes correspond to panel labels in Figure 1; Values of *p* were calculated using two-way ANOVA with main effects of *R. dorsalis* (viruliferous vs. nonviruliferous), rice (virus-infected and uninfected), and their interaction. Values of *p* in boldface are significant at the level of 0.05.

### Viral Infection Affects the Feeding Behavior of *R. dorsalis*

**Figure 3:**
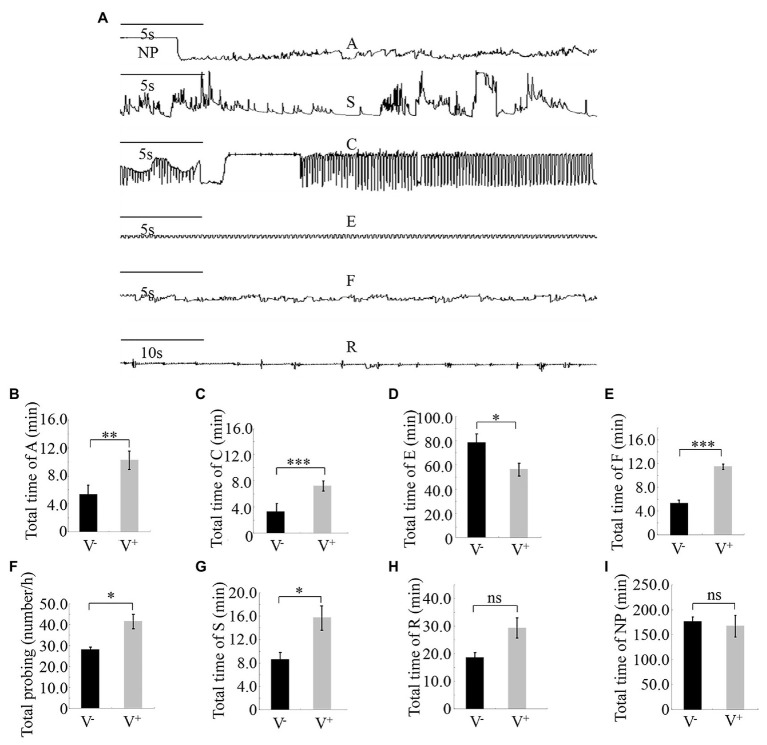
Feeding behavior of viruliferous or nonviruliferous *R. dorsalis* on rice plants, as detected by electrical penetration graph (EPG) assay. **(A)** Characterization of the EPG waveforms produced by feeding *R. dorsalis* on rice plants, including waveforms NP, A, S, C, E, F, and R. NP, not probing. A, stylet movement in the tissue. S, intracellular salivation in mesophyll cells. C, active ingestion from the phloem. E, passive phloem sap ingestions. F, obstacle waveform. R, rest waveform. **(B–I)** Viral infection of insect vectors affected the feeding behavior of *R. dorsalis*. The data were electrically recorded during 5 h feeding period for viruliferous or nonviruliferous insects on healthy rice plants. V^−^, nonviruliferous adults feeding on healthy rice; V^+^, viruliferous adults feeding on healthy rice. Bars represent means ± SE. Significant differences in feeding behavior are denoted as *(p, 0.05), **(p, 0.01), or ***(p, 0.001); student’s t-test.

Different feeding activities during insect probing produced distinct EPG waveforms. Characterization of the waveforms produced by *R. dorsalis* feeding revealed three main types of feeding behaviors: non-ingestion probes, short-ingestion probes, and long-ingestion probes (Figure 3A). The waveform NP indicates that *R. dorsalis* stays on the leaf surface without probing or feeding; waveform A demonstrates that the stylet begins to probe between the mesophyll cells in a short duration time; waveform S indicates that the saliva is continuously secreted as the stylet penetrates the epidermal tissue to the phloem; waveform C indicates that the active ingestion before the stylet reaches the phloem; waveform E indicates the passive prolonged ingestion in the phloem; waveform F indicates the obstacle signal induced by the obstruction of mechanical puncture with the stylet in the extracellular or intracellular cell wall, such as mesophyll cells and tubular cells, called sieve tubes; waveform R indicates the insertion of the stylet into the leaf tissue.

Pairwise comparisons of the feeding behavior of viruliferous or nonviruliferous *R. dorsalis* on rice plants revealed that the frequency and mean duration of non-ingestion (waveform A) and active ingestion (waveform C) probes were greater for the viruliferous group than for the nonviruliferous group (Figures 3B,C). Contrarily, the mean duration of individual passive ingestion probes (waveform E) was significantly shorter for the viruliferous group than the nonviruliferous group (Figure 3D). As they fed, viruliferous insects encountered stronger physical barriers, including phloem plugging, than nonviruliferous insects (Figure 3E). Meanwhile, the mean duration of individual salivary secretion (waveform S) and rest (waveform R) probes was significantly longer for the viruliferous group than for the nonviruliferous group (Figures 3F–I). Thus, viral infection potentially prolongs salivary secretion and ingestion probing to facilitate viral release into plant phloem. Also, viruliferous *R. dorsalis* is protected from continuous ingestion of phloem sap from rice plants. These findings demonstrate that viral infection of insect vectors impacts the feeding behavior of *R. dorsalis* to facilitate viral transmission into the plant phloem.

### Viruliferous *R. dorsalis* Induces More Callose Deposition in Rice Plants

**Figure 4:**
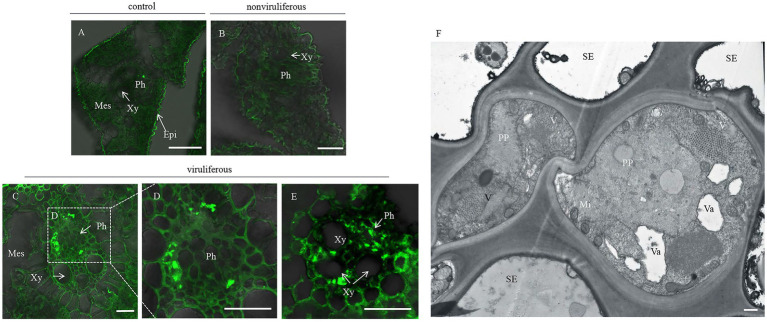
Rice gall dwarf virus distribution in rice phloem after infested by viruliferous *R. dorsalis*. **(A–E)** Immunofluorescence detection of RGDV in the cross-sections prepared from leaf phloem infested with nonviruliferous or viruliferous *R. dorsalis*. Nonviruliferous **(B)** or viruliferous **(C–E)** *R. dorsalis* adults were fed on healthy rice plants for 3 days, which were then processed for immunofluorescence with RGDV P8 antibody conjugated to fluorescein isothiocyanate (FITC). Non-infested healthy rice leaves served as a control. Xy, xylem; Ph, phloem; Mes, mesophyll. **(F)** The distribution of RGDV particles in rice phloem parenchyma was observed by electron microscopy. V, RGDV virions; PP, phloem parenchyma; SE, sieve elements; Va, vacuole; Mi, mitochondrion. Scale bars: 25 μm **(A–E)** and 500 nm **(F)**.

**Figure 5:**
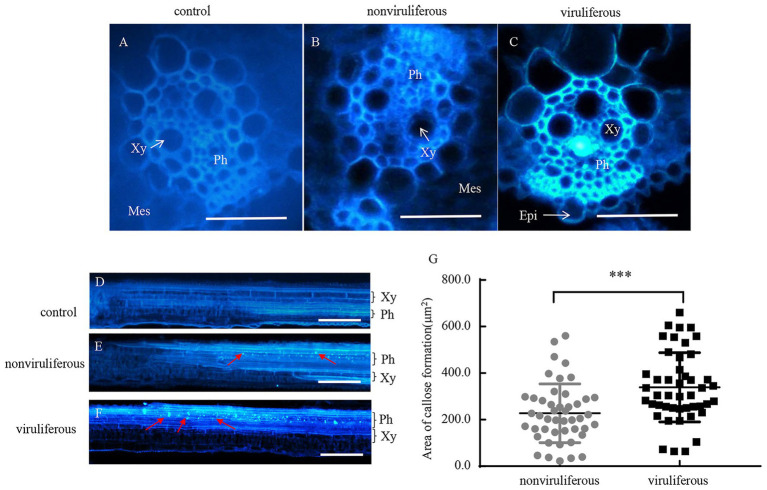
Infection of RGDV by *R. dorsalis* activated the callose deposition in rice phloem. **(A–F)** Induced callose deposition in the phloem depicted by bright blue fluorescence in the cross-sections **(A–C)** and longitudinal-sections **(D–F)** prepared from leaf phloem infested with nonviruliferous or viruliferous *R. dorsalis*. The sample without insect infestation served as the control. The thin sections were stained with 0.1% aniline blue at 3 days after *R. dorsalis* feeding and examined under a fluorescence microscope. Xy, xylem; Ph, phloem; Mes, mesophyll; Epi, epidermis. Arrows indicate the bright blue fluorescence. Scale bars: 50 μm. **(G)** The average areas of sieve plates with callose deposition in nonviruliferous or viruliferous *R. dorsalis*-infested leaf sheaths were counted using Image J. Error bars denote ± SE of sieve plates with callose deposition observed in 46 cross-sections. Significant differences in callose deposition are denoted as ^***^(*p*, 0.001); student’s *t*-test.

Phloem plugging, characterized by callose deposition on sieve plates in plants, is one important mechanism that prevents insects from ingesting phloem sap (Cheng et al., 2013). In rice plants, RGDV infection is restricted to phloem sieve cells (Chen et al., 2016). Immunofluorescence assay revealed that the major outer capsid protein P8 of RGDV appeared in the sieve tube cells of rice phloem to establish initial infection as early as 3 days after viruliferous leafhoppers feeding (Figures 4A–E). RGDV virions were clearly observed in the parenchyma of rice phloem by electron microscopy (Figure 4F). Since viruliferous *R. dorsalis* is protected from continuously ingesting phloem sap from rice plants, viral infection may unplug sieve tube occlusions in rice phloem. The callose deposition on the sieve plates during the feeding of *R. dorsalis* on rice plants was observed by staining with 0.1% aniline blue and examined under a fluorescence microscope. However, little or no callose deposition was found on the sieve plates in the leaf sheaths during feeding by nonviruliferous insects (Figures 5A,B,D,E). Upon infestation of plants with the viruliferous insects, more calloses were deposited on the sieve plates at the point where stylets had been inserted (Figures 5C,F). The sieve plates infested with the viruliferous insects emitted stronger fluorescence than those infested with the nonviruliferous insects (Figure 5G). Thus, viruliferous *R. dorsalis* feeding induced more callose deposition on the sieve plates of rice leaves than nonviruliferous insects.

**Figure 6:**
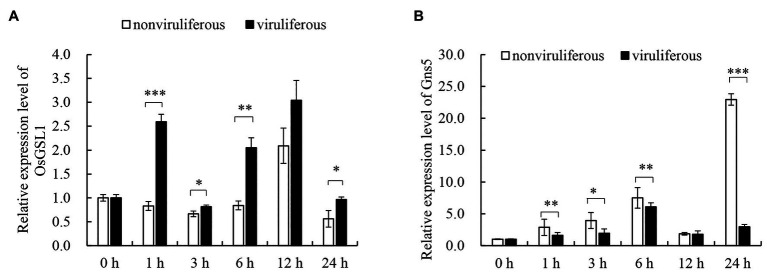
Real-time RT-PCR assay of the callose synthase and callose hydrolyzing genes in response to *R. dorsalis* feeding. The callose synthase-encoding gene GSL1 **(A)** and callose hydrolase-encoding gene Gns5 **(B)** were analyzed. Total RNA was extracted from rice leaf sheaths after different *R. dorsalis* feeding times. Expression of genes was quantified relative to the value obtained from 0 h samples (*R. dorsalis*-free plants). Each bar represents the mean ± SE of three replicates. Each RNA sample was extracted from approximately 100 mg of fresh leaf sheaths of five rice plants. Rice actin gene was used as a reference control. Significant differences in gene expression are denoted as ^*^(*p*, 0.05), ^**^(*p*, 0.01), or ^***^(*p*, 0.001); student’s *t*-test.

The dynamic regulation of callose deposition is driven by callose synthase and hydrolase (Yang et al., 2017). To evaluate the associated mechanisms for the differential callose deposition during the feeding of the nonviruliferous or viruliferous insects on rice plants, the expression of callose deposition-related genes was detected using RT-qPCR assay. The feeding of viruliferous *R. dorsalis* induced higher expression of callose synthase gene OsGSL1 and lower expression of callose hydrolase gene Gns5 in rice plants than nonviruliferous insects (Figures 6A,B). We concluded that the viruliferous insect feeding potentially induces more callose deposition on the sieve plates of rice leaves by activating callose synthase and hydrolase.

### Viruliferous *R. dorsalis* Induces the Increased Cytosolic Ca^2+^ Level in Rice Plants

**Figure 7:**
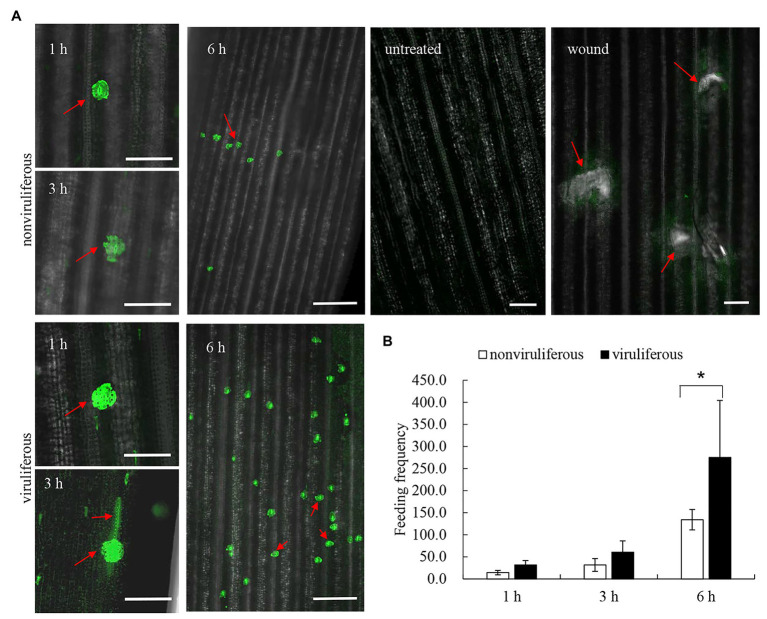
Viruliferous *R. dorsalis* induced the elevated cytosolic Ca^2+^ level in the rice plant. **(A)** Fluochemical intracellular Ca^2+^ determination in leaves infested by viruliferous or nonviruliferous adults of *R. dorsalis*. The green fluorescence refers to the binding of Fluo-3 AM with Ca^2+^. A portion of rice leaf incubated with 5 μM Fluo-3 AM solution was infested by viruliferous or nonviruliferous adults for 1, 3, and 6 h. The sample without insect infestation served as the control. Untreated and wound-treated rice plants were used as controls. Scale bars: 100 μm. Arrows indicated the feeding sites. **(B)** Statistical analysis of feeding sites in infested-leaves by viruliferous and nonviruliferous adults. Significant differences of feeding sites were indicated with ^*^(*p*, 0.05); student’s *t*-test.

Callose deposition, a type of phloem plugging, is potentially induced by the transient Ca^2+^ in plant cytoplasm as the insect feeds on plants (Cheng et al., 2013). We explored whether *R. dorsalis* feeding could influence the cytosolic Ca^2+^ contents in rice plants. The cytosolic Ca^2+^ contents in rice plants that had been fed with viruliferous or nonviruliferous individuals for 1, 3, or 6 h were evaluated using Fluo-3 AM as the Ca^2+^-selective fluorescent indicator. At the viruliferous *R. dorsalis* feeding sites, the adjacent fluorescence intensity was higher than that by nonviruliferous *R. dorsalis* (Figure 7A). This demonstrated that the cytosolic Ca^2+^ contents at viruliferous *R. dorsalis* feeding sites were higher than those at nonviruliferous *R. dorsalis* feeding sites (Figure 7A). Further statistical analysis revealed that viruliferous *R. dorsalis* had more feeding sites than nonviruliferous *R. dorsalis* (Figure 7B).

## Discussion

Vector-borne phloem-inhabiting viral pathogens cause unprecedented economic losses globally and represent some of the most challenging pests to understand due to their specialized feeding strategies (Stafford et al., 2011; Jiang et al., 2019). Understanding the successful transmission mechanism of phloem-inhabiting viruses by piercing-sucking insects into phloem cells still poses a great challenge to researchers. Herein, we use the leafhopper-borne phloem-inhabiting RGDV to investigate the above important issue. Effective transmission of insect-borne plant viruses into plant phloem is closely related to the active insect feeding behavior (Jimenez et al., 2018). We find that nonviruliferous individuals of *R. dorsalis* prefer to feed on and are attracted to RGDV-infected rice plants compared to viruliferous individuals. Attracting more nonviruliferous insects to feed on infected plants potentially promotes viral acquisition and transmission. Of note, RGDV infection is thought to regulate the metabolism of rice secondary substances such as terpenoids, phenols, etc., which consequently may enhance the olfactory attraction to *R. dorsalis*. These findings imply that RGDV could alter plant host preference of insect vectors by changing the plant volatiles to promote viral spread to new hosts.

More importantly, viruliferous insect feeding may manipulate the host phenotypes directly to promote viral spread to new hosts. The piercing-sucking insects use stylets to suck plant phloem sap, and saliva is secreted during insect probing, penetration, and ingestion (Sharma et al., 2014). The infectious virions are injected into the rice phloem *via* the saliva during *R. dorsalis* feeding, after which the initial infection is robustly established in the host phloem. EPG assay demonstrates that viruliferous insects encounter stronger physical barriers such as phloem plugging than nonviruliferous insects during feeding. Viral infection of *R. dorsalis* potentially prolongs the duration of salivary secretion and ingestion probing. Following the EPG assay test, we further demonstrate that viruliferous *R. dorsalis* feeding induces more feeding sites and total probing number. Viruliferous insects spend more time resting or moving about on rice plants. Such observations imply that viral infection strongly affects the feeding behavior of *R. dorsalis*, which promotes the secretion of more infectious virions from the salivary glands into rice phloem. In consequence, infectious virions secreted from the salivary glands of insect vectors are successfully transmitted to plant phloem.

Furthermore, we find that viruliferous *R. dorsalis* feeding induces the deposition of more calloses on sieve plates of rice phloem than nonviruliferous insects. Callose deposition on sieve plates in plants is one of the most crucial defense mechanisms against insects ingesting phloem sap (Cheng et al., 2013). Based on our findings, viruliferous *R. dorsalis* encounters a stronger physical barrier of callose deposition than nonviruliferous insects, which prevents insects from continuously ingesting phloem sap. Likewise, in rice plants harboring brown planthopper resistance genes, callose deposition on the sieve plates occludes the sieve tubes; thus, the continuous feeding by planthoppers is directly impeded (Du et al., 2009). Of note, viruliferous *R. dorsalis* would probe more frequently and secrete more saliva on rice plants, promoting viral transmission. In response, the feeding by viruliferous *R. dorsalis* induces the expression of high level of callose synthase gene OsGSL1 and low level of callose hydrolase gene Gns5, which potentially promotes callose deposition in rice phloem cells. When the rice plants were infested for 24 h, the induction of callose hydrolase expression by nonviruliferous *R. dorsalis* is much higher than callose synthase activation by viruliferous *R. dorsalis*. This might indicate suppression of plant anti-defense reactions by nonviruliferous *R. dorsalis*, but not by viruliferous *R. dorsalis*. Ultimately, viruliferous *R. dorsalis* would probe more frequently and secrete more saliva on rice plants to promote viral transmission. Cheng et al. (2013) found that Ca^2+^ accumulation in sieve cells regulated sieve plate occlusion through callose sealing. In the present experiment, viruliferous *R. dorsalis* feeding significantly elevates cytosolic Ca^2+^ accumulation in rice and potentially induces strong callose deposition. Interestingly, aphids secrete CBP in watery saliva to prevent sieve cell occlusion (Will et al., 2007; Atamian et al., 2013; Wang et al., 2015). Similarly, in the saliva of brown planthopper, CBP is secreted into rice cells to decrease rice cytosolic Ca^2+^ accumulation in rice cells (Ye et al., 2017). Therefore, we deduce that the secretion of CBP from salivary gland of viruliferous *R. dorsalis* is decreased, and thus the cytosolic Ca^2+^ accumulation level is increased to promote sieve cell occlusion. In conclusion, for the first time, the present study demonstrates that the delivery of a phloem-limited virus into the plant phloem by piercing-sucking insects activates the defense-associated callose deposition to enhance viral transmission.

## Data Availability Statement

The original contributions presented in the study are included in the article/supplementary material, further inquiries can be directed to the corresponding author.

## Author Contributions

GY and TW designed the experiments and wrote and revised the manuscript. WW and GY conducted the experiments. All the authors contributed to the article and approved the submitted version.

### Conflict of Interest

The authors declare that the research was conducted in the absence of any commercial or financial relationships that could be construed as a potential conflict of interest.
